# Sex, Genes, and Traumatic Brain Injury (TBI): A Call for a Gender Inclusive Approach to the Study of TBI in the Lab

**DOI:** 10.3389/fnins.2021.681599

**Published:** 2021-05-05

**Authors:** Kelli A. Duncan, Sarah Garijo-Garde

**Affiliations:** ^1^Department of Biology, Vassar College, Poughkeepsie, NY, United States; ^2^Program in Neuroscience and Behavior, Vassar College, Poughkeepsie, NY, United States

**Keywords:** TBI, transgender, gender, sex differences, hormones

## Introduction

Traumatic Brain Injury (TBI) is a leading cause of morbidity and moribundity in the United States (Bruns and Hauser, 2003; Corrigan et al., 2010). A number of factors including sex influence TBI clinical outcome. Both clinical and lab studies show a clear influence of sex on TBI outcome. However, whether this is mediated by hormones, genes, or both is still under debate (Gupte et al., 2019; Ma et al., 2019; Mikolić et al., 2020). The majority of research focuses on factors of endogenous hormone signaling (release and reception) in natal males (Slewa-Younan et al., 2004; Dubal et al., 2006; Herson et al., 2009; Griesbach et al., 2015; Clevenger et al., 2018; Mollayeva et al., 2018; Späni et al., 2018; Ma et al., 2019). This excludes natal females and both males and females taking exogenous hormones for hormone replacement (HRT) or cross sex hormone therapy (CSHT) as part of a gender confirming therapy (Mollayeva et al., 2018; Späni et al., 2018; Ma et al., 2019; Giordano et al., 2020; Biegon, 2021). While transgender and gender non-conforming (TGNC) individuals make up 0.3–0.5% of the global population, they are affected by violence at higher rates compared to cisgender individuals (Jauk, 2013). Despite these higher rates of violence and increased risk of TBI, the TGNC population remains understudied in the TBI field (Safer et al., 2016). This bias extends to healthcare settings where many TGNC individuals face significant barriers to obtaining high-quality, compassionate medical care at primary care facilities, especially in emergency rooms, where most TBIs are diagnosed (Sanchez et al., 2009; Porter et al., 2016; Reisner et al., 2016; Safer et al., 2016; Dickey and Singh, 2017).

Research regarding transgender health has dramatically increased, yet there is still significant room for improvement as TGNC individuals are at an increased risk for several health issues (Reisner et al., 2016; Ackerley et al., 2019; Neblett and Hipp, 2019; Yeung et al., 2019; Wiepjes et al., 2020). A gender inclusive approach in biomedical research is vital to our understanding and treatment of TBI. The aim of this paper is to call upon lab-based investigators to approach the study of TBI and also biomedical research in a gender inclusive manner.

## Sex, Gender, And Tbi: Beyond The Binary

TBI has a biphasic response. While the primary insult is often short in duration, the secondary phase can linger for hours to weeks after the initial injury. Like the primary injury, if untreated, this secondary phase can lead to a manifestation of clinical or behavioral symptoms, including death (Lenzlinger et al., 2001; Bramlett and Dietrich, 2007; Maas et al., 2008). The majority of research focuses on both decreasing the chance of injury and decreasing the secondary effects of that injury (Fitch et al., 1999; Day et al., 2013; Kim et al., 2015; Ripley et al., 2020; Bourgeois-Tardif et al., 2021). Severe symptoms include long-term cognitive or behavioral deficits, while moderate to mild symptoms include headaches, dizziness, nausea, and short-term amnesia (Prins et al., 2013). Additionally, if untreated, these long-term effects of TBI can result in increased risk of neurodegenerative diseases such as Alzheimer's disease, chronic traumatic encephalopathy, and Parkinson's disease (Lye and Shores, 2000; McKee et al., 2009; Hutson et al., 2011). Treatment of secondary injuries is complex, as there are a multitude of neurobiochemical and metabolic pathways that are activated across multiple time scales and can differ depending on sex (Prins et al., 2013; Saldanha et al., 2013; Rahimian et al., 2019; Mikolić et al., 2020).

Hormone production or availability likely contribute to TBI outcome which results in females largely being reported to be more resilient than their male counterparts (Mollayeva et al., 2018; Ma et al., 2019; Rubin and Lipton, 2019). Furthermore, when comparing prepubescent, premenopausal, and post-menopausal women, premenopausal, and pubescent females generally have lower rates of mortality and better prognoses than older, premenopausal adults (Du et al., 2004; Ley et al., 2013; Albrecht et al., 2016; Ranganathan et al., 2016; Ma et al., 2019). These conclusions are supported by studies directly examining the role of estrogens, progesterone, androgens, and their metabolites following TBI. The studies have identified these steroids via activation of their receptors (which can vary by sex following injury) as being neuroprotective by preventing the brain from edema, necrosis, apoptosis, and inflammation (Stein and Hoffman, 2003; Bryant et al., 2006; Dubal et al., 2006; Spence and Voskuhl, 2012; Acaz-Fonseca et al., 2016; Brotfain et al., 2016; Duncan and Saldanha, 2020). These effects can occur acutely after the injury, but can have prolonged effects lasting weeks after the initial injury (Suzuki et al., 2007). However, the majority of these studies have focused on endogenous release vs. exogenous therapy and when comparing humans to lab models, we can see the opposite result (Hall et al., 2005; Stein, 2015; Gupte et al., 2019).

### How to Study TBI Through a Transgender Lens?

The process of transitioning is complex and can be heavily individualized, which partially explains some of the difficulties in developing a lab-based model. Despite the limited information regarding the development of human gender identity, there has been significant progress in using animal models to demonstrate the neurodevelopment pathways leading to sex differences in brain and behavior (Joel and McCarthy, 2017; Choleris et al., 2018; Theisen et al., 2019). From these studies, three main factors: environment, genes, and hormones, have all been identified as mechanisms key to understanding human gender identity.

Environment (social or physical) plays a major role in the development of human gender including transgender identity by directly or indirectly (epigenetics) altering gene expression and behavior (Szyf et al., 2008; Arnold, 2017). However, the role of the environment is difficult to model in non-human subjects; and therefore, we will focus primarily on the other two factors identified (genes and hormones).

### Factors Affecting TBI Outcome in TGNC Populations

#### Genes

A number of studies have suggested a genetic contribution to the development of transgender identity (Lippa and Hershberger, 1999; Bentz et al., 2007, 2008; Fernández et al., 2015; Smith et al., 2015; Fisher et al., 2018; Polderman et al., 2018; Foreman et al., 2019). Specifically, twin studies have found heritability anywhere between 38–47% in adolescent natal females and 25–43% in adolescent natal males, while these numbers decrease to 11–44% and 27–47% in adults (Fisher et al., 2018; Polderman et al., 2018; Theisen et al., 2019). A number of genes were identified from these studies, many of which were also previously identified in studies of sexual differentiation in animal models. These include COMT, PIK3CA, RYR3, SRD5A2, STS, and SULT2A1, as well as variants of genes coding aromatase, androgen receptor (AR), estrogen receptors (ER) α & β, and 17α-hydroxylase (Fernández et al., 2015, 2018; Smith et al., 2015; Yang et al., 2017; Fisher et al., 2018; Foreman et al., 2019; Theisen et al., 2019). In terms of sex differences in TBI, we have also identified differences in a number of these genes as well, including PIK3C, SULT2A1, aromatase, AR and ERs, and 17 hydroxylase (Garcia-Segura et al., 2003; Duncan and Saldanha, 2011, 2013; Saldanha et al., 2013; Pedersen et al., 2018; Cook et al., 2020; Duncan, 2020). Suggesting that these genes may have variations in their response following TBI and can serve as first candidates for examining differences in gene expression. For example, ERα mediates the estrogenic neuroprotective effects of TBI (Dubal et al., 2006; Duncan and Saldanha, 2020) and TGNC individuals have differences in constitutive receptor expression or different polymorphisms that may affect their ability to activate these neuroprotective pathways (Fernández et al., 2018). Discerning how these receptors and genes may change in TGNC individuals is key to our understanding of their activation following TBI.

Furthermore, the use of the four core genotype mouse model which uncouples chromosomal (X, Y) effects from gonadal influence along with the XY^*^ mice could be a powerful tool in determining the chromosomal/genetic contributions during TBI (Arnold and Chen, 2009; Corre et al., 2016; Arnold, 2020). Comparison of XX and XY mice with the same type of gonads, but different sex chromosomes can help in determining the role of sex linked genes vs. hormone availability. While not fully a model for TGNC populations, this is a powerful tool for determining the relative contribution of a sex difference. Currently, two studies have examined TBI using one of these models and found that in young animals, hormones and not chromosomes shaped response; however, this was reversed in aged populations (Manwani et al., 2015; McCullough et al., 2016). More work is on-going to determine the differences in gene expression following TBI, and if these differences are the same between cis and transgender individuals. The use of these two powerful models can help to determine if a sex difference in TBI are mediated by chromosomes, hormones, or both, especially when paired with cross hormone therapies.

#### Hormones

Steroid hormone levels and receptors are markedly different in natal males and females throughout most of their lifespan and clearly play a role following TBIs (Arnold, 2017; Gölz et al., 2019; Giordano et al., 2020). In terms of TGNC individuals, life-long hormonal therapy is often a key component of their transition and can be implemented as early as adolescence (Deutsch et al., 2015; Hembree et al., 2017; Nguyen et al., 2018; T'Sjoen et al., 2019). One major issue with studying individuals that are currently transitioning or have transitioned is identifying the specific medical plan used to transition. A number of various plans are used (Table 1), for both medical and personal reasons, and thus modeling can become difficult to mimic exactly (Feldman and Safer, 2009; Hembree et al., 2009; The World Professional Association for Transgender Health, 2012; American Psychological Association, 2015; Unger, 2016; Funabashi et al., 2018; Defreyne and T'Sjoen, 2019; Hamidi and Davidge-Pitts, 2019). It is important to note that many of the hormones used for transitioning differ to what are commonly used in the lab, specifically in terms of long-term use and “stacking” of multiple drugs. What we currently know about exogenous hormones and neural damage and recovery comes from teasing out the various contributions of a transition plan, but more research is needed to combine all of these components into a comprehensive model of a transitioning or transitioned individual.

**Table 1:** Drugs used for gender affirming hormonal treatments in TGNC individuals.

	**Drug**	**Common drug name(s)**	**Route of administration**	**Proposed dosage with frequency**	**Blood levels (in humans)**
Trans-masculine (FtM)	Testosterone Undecanoate (UK) or Testosterone Enanthate (US) or Testosterone cypionate (US)	• Andriol® • Delatestryl® • Depo®-Testosterone • Aveed® • Androgel®, Androderm®	Oral, Subcutaneous, Intramuscular, Transdermal	• Undecanoate: 160–240 mg/day • Enanthate, cypionate: 20–100 mg/week • Transdermal: 2.5–10 mg/day	Testosterone: 300–1,000 ng/dL
	Progesterone (optional)	• Provera®	Oral	• 12.5 mg/daily	
Trans-feminine (MtF)	Estradiol or Estradiol valerate or Estradiol cypionate	• Depo®-Estradiol, Depofemin®, Estradep® • Delestrogen®, Progynon Depot®, Progynova®	Oral, Subcutaneous, Intramuscular, Transdermal	• Estradiol: 2–6 mg daily • Estradiol valerate: 2–20 mg/2weeks • Estradiol transdermal: 0.025–0.2 mg/daily	Estrogen: 100–200 pg/mL
	Anti-Androgens: Progesterone Spironolactone Histrelin implant	• Provera® • CaroSpir®,Aldactone® • Vantas®, Supprelin LA®	Oral, implant	• Progesterone: 25–50 mg PO daily • Spironolactone: 100–300 mg PO daily • Histrelin: 3.75 mg monthly	Testosterone: <50 ng/dL
Puberty blockers	GnRH analogs/agonists: Leuprolide acetate Histrelin	• Lupron Depot® • Vantas®, Supprelin LA®	Subcutaneous, Intramuscular, Implanted pellet	• Lupron Depot: 7.5 mg/monthly • Histrelin: 3.75 mg monthly	Peak LH <4 mIU/mL after GnRHa stimulation.

#### Trans-masculine

TBI has historically been viewed as a problem that predominantly affects natal males, as they are both more likely to receive TBIs and have less of the circulating neuroprotective steroids: estrogen and progesterone (Späni et al., 2018; Gupte et al., 2019; Rubin and Lipton, 2019; Mikolić et al., 2020). When comparing age-matched natal males and females, younger females appear to be protected against neuronal damage, suggesting that androgens may not be advantageous following injury (Dubal and Wise, 2002; Gupte et al., 2019). However, this is complicated by research that shows that males with lower testosterone have worse clinical outcomes than males within normal ranges, suggesting that while testosterone isn't detrimental in males, that other steroids may be more beneficial. This is supported by studies of natal males given testosterone for myelin repair for relapsing-remitting Multiple Sclerosis that show a significant increase in neuroprotection over controls (Kurth et al., 2014). More research is necessary to further identify the role of testosterone following TBI.

In trans-masculine procedures, hormone therapy is sometimes paired with removal of the uterus and ovaries (hysterectomy and oophorectomy) via gender confirmation surgery (Coleman et al., 2012; American Psychological Association, 2015). Ovary removal has profound effects on both circulating hormone levels and TBI outcome. Cisgender women undergoing oophorectomy show lower levels of estrogen than age-matched women experiencing natural menopause (Korse et al., 2009; Perera et al., 2013; Orozco et al., 2014). Post-menopausal cisgender women, characterized by decreased circulating estrogens and progestins, show worse outcomes than premenopausal females, but better than age-matched natal males (Niemeier et al., 2013). This suggests that removing circulating hormones affects TBI severity and could potentially make FTM individuals more susceptible to neurodegeneration. Furthermore, when studying risk of neural damage, epidemiological evidence clearly shows that sex and estrogen levels are important factors in long-term outcome (Rocca, 2017; Bazzigaluppi et al., 2018). Studies of ovariectomy prior to TBI in rats showed larger areas of damage compared to intact females and thus worse outcomes (Bramlett and Dietrich, 2001). Together, these data suggest that although steroid hormones may be protective, their sudden withdrawal either before or after injury may be a key factor contributing to worse outcomes in individuals assigned female at birth (Wunderle et al., 2014). Put together, these data suggest that transgender males that elect for gender affirmation surgeries may be more susceptible to negative outcomes of TBI. However, more research is needed to understand how this can be alleviated.

#### Trans-feminine

If natal females are indeed better protected from TBI, then individuals undergoing trans-feminine transition may see better outcomes following hormonal transition as they typically take exogenous estrogen and progesterone, as well as anti-androgens. Typically, younger-aged cisgender women appear to be protected against neuronal damage, compared with cisgender men, but lose this advantage in their post-menopausal years (Niemeier et al., 2013; Ranganathan et al., 2016). A typical cycling female shows monthly variation in both estrogen and progesterone signaling. By using this natural variation in estrogen and progesterone response, we have been able to identify the relative contribution of estrogen and progesterone following TBI. Cisgender women in the luteal phase of their menstrual cycle, in which progesterone is highest, had worse outcomes than those in the follicular phase, in which progesterone is initially low and can therefore not decrease significantly (Wunderle et al., 2014). However, exogenous progestin use from oral contraceptives leads to better outcomes than controls in individuals assigned female at birth (Wunderle et al., 2014). The potential use of steroids in natal men following TBI has led to mixed results [see Späni et al. (Späni et al., 2018) for review]. For estrogens, the negative effects (cardiovascular disease and breast cancer) associated with short-term or long-term use overshadow any potential neuroprotective effects (Späni et al., 2018). Progesterone, however, has been included as a treatment option in two large Phase III trials [ProTECT and SYNAPSE (Wright et al., 2007; Stein, 2015)]. Results from these two trials were not conclusive as some saw no difference in cisgender males, while a small subset saw a slight improvement in them (Lu et al., 2016; Späni et al., 2018). Specifically, sex and hormones present at the time of injury were cited as factors that mediated the effectiveness of estrogens and progesterone following injury (Stein, 2015; Stein et al., 2016; Späni et al., 2018).

Individuals undergoing trans-feminine gender affirming surgeries sometimes remove the testes, a significant source of testosterone (Coleman et al., 2012; American Psychological Association, 2015). Paired with antiandrogen hormonal treatments, this significantly removes the amount of androgens in circulation. Therefore, one would assume that these individuals would show better responses to TBI than transgender men. However, inherent differences in gene expression in neuroinflammatory pathways or vasculature could lead to differences in response to TBI.

The bulk of research into hormone use following TBI has been done in adult or possibly aged populations. However, a significant number of individuals begin this sort of transition in early adulthood or adolescence (Smith et al., 2001; Menvielle and Gomez-Lobo, 2011; Olson, 2016). Currently, there is relatively little research on the role of cross sex hormone therapies in younger populations and nothing known about this topic in TBI research. TBI is the leading cause of death and disability in children (Ley et al., 2013; Araki et al., 2017), and children and adolescents diagnosed with a TBI are at higher risk of being diagnosed with a central endocrinopathy (Ortiz et al., 2020). These TBI induced differences in endocrine function are varied, but have been known to increase disturbances in puberty (Auble et al., 2014). To date, no studies have examined how use of CSHT could affect these pediatric outcomes and or recovery from TBI specifically.

## Discussion

Although we have placed a great emphasis on the lack of research in TGNC individuals, sex/gender as a biological variable is underrepresented in TBI research and requires further analysis. The increase in the number of individuals undergoing hormonal or surgical treatment to aid in gender transition calls for a substantial increase in TBI research in these underserved and marginalized populations. There is a need for evidence-based guidelines, common hormone plans, and clinically translatable diagnostic and prognostic models. Furthermore, expanding our knowledge of how exogenous hormone use affects TBI could have profound effects not only on TGNC populations, but also cisgender males and females. Such work and studies will not only help to develop better treatment options for those identifying as TGNC, but will also create a conceptual framework which can be used to extrapolate to others undergoing hormone replacement or depletion in the future.

## Author Contributions

KD and SG-G contributed equally the development and authoring of this manuscript.

## Conflict of Interest

The authors declare that the research was conducted in the absence of any commercial or financial relationships that could be construed as a potential conflict of interest.
